# Is the Field Test of FUT-SAT a Better Experimental Design to Identify the Specific Characteristics of Tactical Performance according to Youth Male Soccer Players' Positional Roles?

**DOI:** 10.5114/jhk/170044

**Published:** 2023-10-11

**Authors:** Juan Ciro-Cardona, John Marulanda-Tabares, Felipe Moniz, Wilder Valencia-Sánchez

**Affiliations:** 1 Instituto Universitario de Educación Física y Deporte, Universidad de Antioquia UDEA, Medellín, Colombia.; 2 Centro Universitário de Formiga, Minas Gerais, UNIFOR-MG, Brasil.

**Keywords:** youth sport, playing position, team sports, game performance, tactical behaviour

## Abstract

This study aimed to compare soccer players’ tactical performance according to their positional roles in the field test of FUT-SAT. The sample consisted of 54 male players from elite youth clubs (Medellín-Colombia), U16 and U17 categories. Participants trained five times per week (Tier 3). We used the System of Tactical Assessment (FUT-SAT), which evaluates tactical behaviour and performance through core tactical principles of soccer. The field test is structured in a numerical configuration of a goalkeeper + 3 players vs. 3 players + a goalkeeper played during 4 min on a playing field 36 m long by 27 m wide. All teams were structured with one player in each positional role (one defender, one midfielder, and one forward).To determine the differences between the positional roles, the following factors were analysed: the number of actions, the percentage of correct actions, the place of action related to the principles, and the Tactical Performance Index of core tactical principles. A total of 2891 tactical actions distributed in nine games were analysed. A Kruskal-Wallis test for the independent groups (defenders, midfielders, and forwards) or a one-way ANOVA was used. There was no difference in soccer players’ tactical performance and behaviour between players of different positional roles in the field test of FUT-SAT.Therefore, teams need to be evaluated with the system of play with players in all field sectors in medium or large sided games. The system of play can be chosen according to the category given that competitions are held in reduced spaces and with fewer players

## Introduction

Small-Sided Games (SSGs) are play-form activities organized on the field where players perform their actions in smaller spaces, areas, and goals than in the match play (Ford et al., 2010). According to the design of SSGs, it is possible for players to adjust their behaviour and improve their performance in a context that presents similarities with the match play, such as high variability and random practice (Williams and Hodges, 2005). Thus, SSGs are an important training method that allows the development of technical, tactical, physical, and psychological skills with the purpose to enable players to play efficiently and effectively in the match play regardless of their age or category (Aguiar et al., 2012; Sarmento et al., 2018; Szwarc et al., 2015). In this way, assessment of soccer players in SSGs is necessary to understand the specificity of each design, being a useful tool to improve the training sessions (Serra-Olivares et al., 2016). Moreover, the assessment of soccer players’ performance in SSGs is also used to identify their skill proficiency through technical performance in order to help coaches identify the potential of each player and select the best players for each team (Bennett et al., 2018; Dolański et al., 2018; Fenner et al., 2016).Besides these possibilities, assessment of soccer players’ tactical performance in SSGs provides information on the management of their tactical actions on the field (Gréhaigne et al., 1999). Each player in SSGs is part of a positional role, which are categories that regulate players’ playing positions within a system of play in each field sector and are divided into defenders, midfielders, and forwards. Each of them performs offensive and defensive tactical actions throughout the field with common goals (Hewitt et al., 2016). These tactical actions, based on core tactical principles, are performed according to the demands of the game context to generate effective solutions on the field and are related to tactical performance (da Costa et al., 2009; Gréhaigne et al., 1999).

Some researchers assess soccer players’ tactical performance according to their positional role using the field test of System of Tactical Assessment (FUT-SAT) (da Costa et al., 2011). The FUT-SAT is a tool that evaluates soccer players’ tactical performance, based on core tactical principles, in a field test structured in a SSG with the following configuration: a goalkeeper + 3 players vs. 3 players + a goalkeeper (GK + 3 vs. 3 + GK) on a field with dimensions of 36 m x 27 m during four minutes (Costa et al., 2011). This SSG has the minimum structure necessary to identify all core tactical principles of soccer, and the aim of the FUT-SAT is to provide information that reflects tactical performance of players in a match context, like SSGs (González-Víllora et al., 2015). Thus, some studies have compared soccer players’ tactical performance between different positional roles in SSGs.

The results of those studies have shown significantly higher values of tactical performance of core tactical principles for the following positional roles: midfielders in relation to forwards for the offensive unity in U13 (Padilha et al., 2013), full-backs in relation to forwards in U17 in the defensive unity, and total defensive actions (Machado et al., 2019). In addition, midfielders and forwards in relation to defenders for the offensive unity and defenders and forwards in relation to midfielders in defensive coverage in U17 (Rechenchosky et al., 2017). According to researchers, such findings were likely due to the focus on attack rather than defence, which results in passive behaviour in the defensive phase (Machado et al., 2019); the characteristics of the midfielders’ role in movement within space without opponents (Padilha et al., 2013), and the less complex situations in SSGs where players commit fewer errors in tactical actions (Rechenchosky et al., 2017). Although these results show differences between defenders, midfielders, and forwards in terms of core tactical principles in SSGs, these differences were not consistent across studies and concerned only few and different core tactical principles. Thus, it is not possible to identify specific tactical skills through tactical behaviour and tactical performance for players of each positional role in the field test of FUT-SAT, specifically in U17 (da Costa et al., 2011).

In U17, coaches have already defined players’ positional roles. In this particular category, elite soccer players must spend more hours on team and individual practices, mainly considering the tactical aspects of the game, on training according to their demands with the purpose to develop their skills (Côté and Vierimaa, 2014; Ward et al., 2007). Studies on SSGs, especially systematic reviews, have shown that this playing form is relevant for the development of players in their training process because it stimulates changes in their behaviour according to the size and the number of players on the field, demonstrating effects on their physical, technical, and tactical abilities (Clemente et al., 2020; Clemente and Sarmento, 2020;Duda, 2020; Hill-Haas et al., 2011;Radziminskiet al., 2022 ). Moreover, it is necessary to conduct investigations on the structure and configuration of SSGs to indicate possible experimental designs to assess soccer players according to their positional roles with the purpose of understanding their characteristics in a specific role (Serra-Olivares et al., 2016). Thus, to compare soccer players’ tactical behaviour and performance in different positional roles, it is helpful to discuss whether the structure of the field test (SSGs) provides information about positional roles’ demands with regard to their specific tactical skills (Garganta, 2009; González-Víllora et al., 2015).

Thus, it is necessary to conduct further research to confirm whether the structure of the field test of FUT-SAT is an adequate experimental design to identify the specific tactical skills of each positional role. This study aimed to compare youth male soccer players’ tactical performance according to their positional roles in the field test of FUT-SAT.

## Methods

### Design

This paper presents a non-experimental descriptive cross-sectional study (Thomas et al., 2022) in which the number of actions, success percentage, Place of Action related of Principles (PARP), and Tactical Performance Index (TPI) of players by position were described in a single evaluation moment.

### Participants

The sample consisted of 54 male elite players of youth competitive clubs (age = 16.2 ± 0.88 years; body height = 1.73 ± 0.06 m; body mass = 63.54 ± 6.64 kg; fat % = 13.4 ± 4.68; federated experience = 7.91 ± 2.77 years) (Table 1).

**Table 1:** Descriptive statistics of anthropometric variables and age.

Variable (n = 54)	X̄	SD
Age (years)	16.2	0.88
Body height (m)	1.73	0.06
Body mass (kg)	63.54	6.64
Body fat(%)	13.4	4.68
IMC (kg/m^2^)	21.17	1.94
Federated experience (years)	7.91	2.77

BMI = body mass index; SD =standard deviation

**Figure 1:**
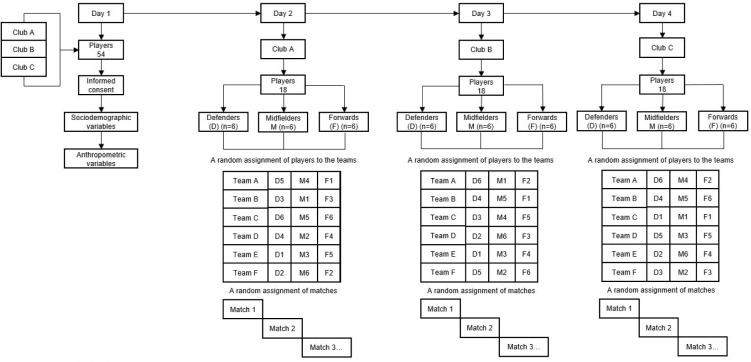
A study timeline.

Participants were selected from one of the three teams that participated in the U16 and U17 Level A of the Antioquia Football League (Colombia) (n = 18 from club A; n = 18 from club B; and n = 18 from club C). This is equivalent to Tier 3, Highly Trained/National Level, in the Participant Classification Framework (McKay et al., 2022). Participants trained five times per week for a period of 90 min, with one competition match on the weekend.This investigation started 13 weeks after the season began, when the first round was completed, but before the commencement of the second round. The assessments took four days. On the first day, assent and informed consent forms were collected, in addition to the sociodemographic and anthropometric variables. On the second, third, and fourth days, tactical performance was evaluated, one day per club, as shown in Figure 1. A tactical performance evaluation was performed after a full recovery (72 h after a match). Assessments took place on Monday, at the training facilities of each cub (A, B, and C) at 9:00 a.m., 12:00 p.m., and 2:00 p.m. with ambient temperatures of 16°C, 25°C, and 26°C, relative humidity of 71%, 48%, and 47%, and a height of 250 MASL, 1495 MASL, and 1495 MASL, respectively. The playing surface was synthetic grass for clubs A and B, and a gravel field for club C. In a previous study, no differences in surface types or tactical performance were observed for the game format GK + 3 vs. 3 + GK for four minutes (Costa et al., 2009). Participants were tactically evaluated and distributed by positional roles, including defenders (n = 18), midfielders (n = 18), and forwards (n = 18). All teams were structured with one player in each positional role (one defender, one midfielder, and one forward). Position stratification was performed by the coach based on the number of minutes played in the competition, who reported it to researchers once they agreed to participate in the study. A total of 2891 tactical actions distributed in nine games were analysed.

The convenience sample was recruited from the three competitive soccer clubs enrolled in the local league. Players met the following inclusion criteria: 1) male, 2) a member of the Antioquia Football League, 3) signed the U16 and U17 category informed consent, and 4) affiliated with the national health system. The exclusion criteria were as follows: 1) medical history of a positive test for the SARS-CoV-2 virus, 2) musculoskeletal injuries of less than two months, 3) uncontrolled refractory disorders, 4) nonattendance on the day of the evaluation, and 5) refusal to perform the test or not finishing the tests due to physical impediments. To carry out the study of the tactical performance index of soccer players by positional roles, a priori calculations of the power of the study were performed; for this purpose, the data were obtained from a study comparing the defensive and offensive tactical performance of U-17 soccer players from different playing positions (Gonçalves et al., 2017), where a mean difference of 1.68 points was found between forwards and defenders. Thus, the probability of making a type II error was calculated for this study with 18 players per position, a mean difference of 32.98 and 34.66, a standard deviation of 5.1, and a group size of 18 participants, with Z = −1.40; *p* = 0.0807, and power 1 − 0.0807 = 0.919. This means a study power of 92%.

### Measures

For this study, variables of age, body height, mass, body mass index (BMI), and the percentage of fat were analysed. An *ad hoc* template was designed for data collection. Body height was measured with a stadiometer (206, Seca, Germany) fixed to the wall with a measurement range of 200 cm and accuracy of one millimetre. Body mass, BMI, and the percentage of fat were evaluated with electrical bioimpedance (HBF-516, Omron, Japan) with accuracy of 2.2% to 3.3% in body fat. The data were collected following the protocol established by Alvero et al. (2009).

The System of Tactical Assessment (FUT-SAT) (Costa et al., 2011) is a tool that evaluates soccer players’ tactical behaviour and performance structured under the core tactical principles of soccer (da Costa et al., 2009). The core tactical principles are divided into five offensive principles (Table 2), i.e., penetration, offensive coverage, mobility, width and length, and the offensive unity, and five defensive principles, i.e., delay, defensive coverage, concentration, defensive balance, and the defensive unity (da Costa et al., 2011, 2009). The TPI was calculated with the following formula: TPI = Σ tactical actions (PP × QP × PA × AO)/number of tactical actions (Costa et al., 2011). The FUT-SAT was validated for content validity, construct validity, and observational reliability with moderate consistency using a minimum Kappa index of 0.79 to 0.99.(Costa et al., 2011).

**Table 2:** Categories, subcategories, variables, and definitions in the observation instrument of the System of Tactical Assessment.

Category	Sub-categories	Variables	Definitions
**Core tactical principles**	Offensive	Penetration	Reducing the distance between the ball carrier and the opponent's goal or baseline.
		Offensive coverage	Offering offensive support to the ball carrier.
		Mobility	Movement of players between the last defender and the goal line.
		Width and length	Movement of players to expand the effective playing space.
		Offensive unity	Offensive advance or support movements of the players in the last line of their own team.
	Defensive	Delay	Actions to slow down the opponent´s attempt to move forward with the ball.
		Defensive coverage	Positioning of the-ball defenders behind the “delayed” player, providing defensive support.
		Balance	Positioning of the off-ball defenders in response to the movements of the attacking team in attempt to establish numerical stability or superiority.
		Concentration	Increased defensive protection around the greatest risk to the goal.
		Defensive unity	Reduction of the effective playing space of the opposing team.
**Place of action related to the principles**	Offensive Midfield	Offensive Tactical Actions	Carrying out offensive tactical actions in the offensive midfield.
		Defensive Tactical Actions	Carrying out tactical defensive actions in the offensive midfield.
	Defensive Midfield	Offensive Tactical Actions	Carrying out offensive tactical actions in the defensive midfield.
		Defensive Tactical Actions	Carrying out tactical defensive actions in the defensive midfield.

Source:da Costa et al. (2011, p. 74).

### Procedures

**Figure 2:**
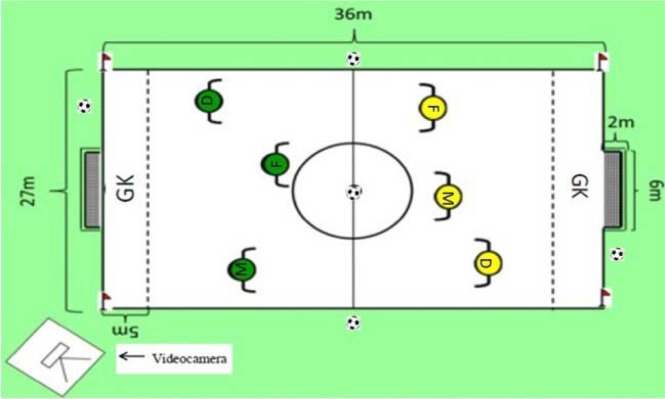
System of tactical assessment; Gk + 3 vs. 3 + GK Gk = goalkeeper; D = defenders; F = forwards. Modified by González-Víllora et al. (2015, p. 14)

The demarcation and delimitation of the space took 45 min per evaluation day. Soccer balls used in the test (Magnum Professional, Golty, Colombia) were calibrated (size = 5, measurement = 66–68 cm, ball weight = 320–390 g, pressure = 0.6–0.8 bar). Participants signed informed consent and assent forms ten days prior to testing. Players were randomly assigned to each team, and each team was randomly assigned to matches. This was performed one week before (Figure 1). Before starting the test, players completed a 20-min warm-up in the following order: joint mobility (five minutes), a general warm-up (eight minutes), and a specific warm-up (seven minutes). While the first match was taking place, the other teams practiced soccer kicks and soccer tennis in the surrounding area. Ball collectors were located on each side of the playing field (Figure 2). The numerical configuration GK + 3 vs. 3 + GK ensured that all the core tactical principles were reached for the field test with duration of four minutes on a playing field 36 m long by 27 m wide (Costa et al., 2011). Players were acquainted with the test three minutes before its application. The test was carried out with all the soccer rules, including the offside rule, because this category competes in the championship in this way. The goalkeeper might only play within the penalty area (5 m) and could not leave this restricted area. After each goal, the ball was to be restarted by the goalkeeper who received it, not from the midfield (González-Víllora and Da Costa, 2015). The teams were randomly selected by a defender, midfielder, and forward (Figure 2). No games were played between Club A, B, and C teams.

### Statistical Analysis

Data normality was checked with the Shapiro-Wilk test. The mean and standard deviation were used to summarize data with a normal distribution, and the median and interquartile range were used to summarize data with a non-normal distribution. The descriptive analysis used the mean and standard deviation for the number of actions, the percentage of correct actions, PARP, and TPI of fundamental tactical principles. To identify possible differences between the positional roles of players, the non-parametric Kruskal-Wallis test was applied alongside the parametric one-way ANOVA with the Tukey's post hoc test. For statistical analysis, a significance level of 5% (*p* ≤ 0.05) was considered. In addition, the statistical program for social sciences (IBM SPSS Statistics 22 for Windows, SPSS Inc., Chicago, Illinois, USA) was used.

### Reliability

After 21 days, a reliability analysis of the data was conducted to corroborate the first data analysis through a test-retest method (Robinson and O’Donoghue, 2007). Thus, 822 tactical actions were reassessed randomly, which represented 28.43% of the sample considering that this percentage was above the reference value recommended (10%) in the literature (Tabachnick and Fidell, 2012). Reliability analysis of the intra-observer and inter-observer evaluations involved the use of the Cohen’s Kappa index. The results of the test-retest method for intra-observer were between 0.860 (SE = 0.120) and 0.96 (SE = 0.009) and for inter-observer between 0.860 (SE = 0.100) and 0.875 (SE = 0.160). Two trained observers participated in this procedure. They analysed the tactical videos for approximately three hours a day, taking breaks and active pauses (15 to 20 minutes) to avoid cognitive fatigue.

### Ethical Aspects

This study was conducted following the regulations of the Colombian Ministry of Health (Resolution 8430 of 1993) (Congreso de la República sobre las Consideraciones Éticas para la Investigación con Seres Humanos., 1993) and the principles of the Declaration of Helsinki for research (World Medical Association, 2013). This study was approved by the ethics committee of the University Institute of Physical Education and Sports of the University of Antioquia (Medellín-Colombia).

## Results

**Figure 3:**
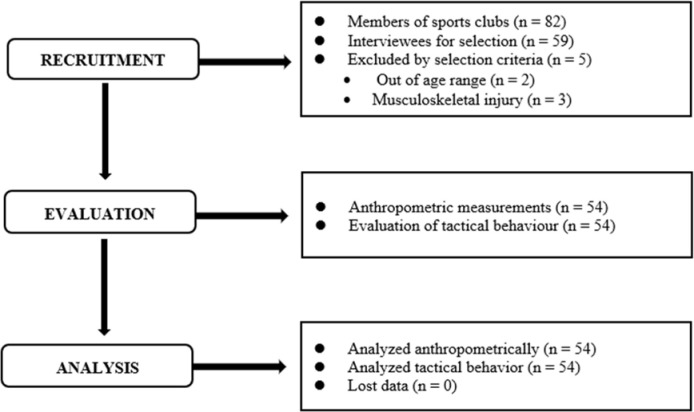
Study participation flow chart.

Figure 3 shows the study participation flow chart. The study sample was initially composed of 82 participants. However, following the inclusion and exclusion criteria, finally fifty-four soccer players were included with 18 players under 16 (33%) and 36 players under 17 (67.7%) (Table 3). A total of 2891 tactical actions (1354 offensive tactical actions and 1627 defensive tactical actions) distributed over nine matches were analysed. Tables 4 and 5 present the mean and standard deviation of the number of actions, the success percentage, PARP, and TPI of players considering their position role, i.e., defenders, midfielders, and forwards. There were no significant differences between players of different positional roles in all core tactical principles within all categories of tactical behaviour.

**Table 3:** Percentage of participation by category.

Category	Frequency	Percentage
**U-16**	18	33.3
**U-17**	36	67.7
**Total**	54	100

**Table 4:** Mean (X̄) and Standard Deviation (SD) of the number of actions and Place of Action Related to the Principles (PARP) of the Positional Roles: Defenders, Midfielders, and Forwards.

Core Tactical Principles	Positional Roles	Number of actions			PARP		
		X̄± SD	CI 95%	*p*	X̄± SD	CI 95%	*p*
**Penetration**	**Defenders**	2 ± 1.74	1.13–2.86	0.935	1.38 ± 1.37	0.7–2.07	0.448
	**Midfielders**	2.11 ± 1.74	1.24–2.97		1.27 ± 1.63	0.46–2.09	
	**Forwards**	2.66 ± 2.05	1.64–3.69		1.61 ± 1.19	1.01–2.2	
**Offensive Coverage**	**Defenders**	6.61 ± 3.82	4.71–8.51	0.35	2.38 ± 1.5	1.64–3.13	0.444
	**Midfielders**	7 ± 2.84	5.58–8.41		3.38 ± 2.25	2.26–4.5	
	**Forwards**	5.77 ± 2.28	4.63–6.91		2.94 ± 2.04	1.92–3.96	
**Mobility**	**Defenders**	1.5 ± 1.38	0.81–2.18	0.915	1 ± 1.08	0.46–1.53	0.570
	**Midfielders**	1 ± 1.08	0.46–1.53		0.61 ± 0.77	0.22–0.99	
	**Forwards**	1.5 ± 1.61	0.69–2.3		1 ± 1.23	0.38–1.61	
**Width and Length**	**Defenders**	10.11 ± 3.81	8.21–12	0.507	3.27 ± 2.46	2.05–4.5	0.789
	**Midfielders**	9.88 ± 4.92	7.44–12.33		2.77 ± 2.26	1.65–3.9	
	**Forwards**	11.77 ± 6.69	8.45–15.1		3.27 ± 2.56	2–4.55	
**Offensive Unity**	**Defenders**	5.22 ± 2.69	3.88–6.56	0.565	2.44 ± 2.52	1.18–3.7	0.606
	**Midfielders**	5.5 ± 3.12	3.94 – 7.05		2.16 ± 1.94	1.19–3.13	
	**Forwards**	4.55 ± 2.5	3.31–5.79		2.55 ± 1.42	1.84–3.26	
**Delay**	**Defenders**	6.5 ± 2.87	5.07–7.92	0.320	3.72 ± 2.13	2.65–4.78	0.459
	**Midfielders**	7.27 ± 2.08	6.24–8.31		3.66 ± 2.35	2.49–4.83	
	**Forwards**	6.11 ± 3.66	4.29–7.93		3.11 ± 2.54	1.84–4.37	
**Defensive Coverage**	**Defenders**	0.83 ± 1.04	0.31–1.35	0.638	0.38 ± 0.77	0–0.77	0.637
	**Midfielders**	1.11 ± 1.18	0.52–1.69		0.5 ± 0.7	0.14–0.85	
	**Forwards**	1.61 ± 2.03	0.6–2.62		0.44 ± 0.98	−0.04–0.93	
**Concentration**	**Defenders**	4.22 ± 3.62	2.42–6.02	0.620	2.55 ± 2.38	1.37–3.74	0.370
	**Midfielders**	4.44 ± 3.01	2.94–5.94		2.83 ± 2.12	1.77–3.88	
	**Forwards**	3.22 ± 2.18	2.13–4.3		1.83 ± 1.58	1.04–2.61	
**Balance**	**Defenders**	5.44 ± 1.97	4.46–6.42	0.236	2.33 ± 1.68	1.49–3.16	0.302
	**Midfielders**	6.05 ± 3.29	4.41–7.69		2.44 ± 2.09	1.4–3.48	
	**Forwards**	7.27 ± 4.11	5.23–9.32		4 ± 3.36	2.32–5.67	
**Defensive Unity**	**Defenders**	13.38 ± 5.4	10.7–16.07	0.526	4.94 ± 3.84	3.03–6.85	0.441
	**Midfielders**	11.61 ± 3.95	9.64–13.57		4.27 ± 2.24	3.16–5.39	
	**Forwards**	12.38 ± 4.59	10.1–14.67		6.05 ± 4.1	4.01–8.09	

CI 95%: 95% confidence interval; *p*< 0.05

**Table 5:** Mean (X̄) and Standard Deviation (SD) of the percentage of correct actions and Tactical Performance Index (TPI) of the Core Tactical Principles of the Positional Roles: Defenders, Midfielders, and Forwards.

Core Tactical Principles	Positional Roles	Percentage of correct actions			TPI		
		X̄± SD	CI 95%	*p*	X̄± SD	CI 95%	*p*
Penetration	Defenders	68.75 ± 34.89	50.15–87.34	0.534	49.71 ± 24.42	36.69–62.72	0.935
	Midfielders	63.54 ± 43.12	40.56–86.52		50.52 ± 25.54	36.91–64.13	
	Forwards	57.22 ± 28.84	41.24–73.19		49.27 ± 24.45	35.73–62.82	
Offensive Coverage	Defenders	81.34 ± 21.67	70.56–92.12	0.826	46.44 ± 10.8	41.06–51.81	0.350
	Midfielders	82.51 ± 19.71	72.71–92.31		50.47 ± 12.35	44.32–56.61	
	Forwards	87.22 ± 13.32	80.59–93.84		49.45 ± 10.19	44.38–54.52	
Mobility	Defenders	43.05 ± 42.91	15.79–70.31	0.960	35 ± 13.96	26.12–43.87	0.915
	Midfielders	40.9 ± 43.69	11.55–70.26		33.97 ± 22.25	19.02–48.92	
	Forwards	38.88 ± 46.78	9.16–68.61		35.27 ± 24.56	19.66–50.88	
Width and Length	Defenders	71.15 ± 21.61	60.4–81.9	0.293	40.24 ± 10.45	35.04–45.43	0.424
	Midfielders	72.71 ± 18.87	63.33–82.1		38.75 ± 8.46	34.54–42.95	
	Forwards	81.65 ± 15.56	73.65–89.65		43.62 ± 9.62	38.67–48.56	
Offensive Unity	Defenders	79.32 ± 20.14	69.3–89.34	0.897	45.08 ± 13.02	38.6–51.56	0.565
	Midfielders	78.39 ± 25.02	65.53–91.26		45.1 ± 15.1	37.33–52.87	
	Forwards	79.25 ± 28.72	64.97–93.54		53.69 ± 21.38	43.06–64.33	
Delay	Defenders	47.59 ± 26.3	34.51–60.68	0.253	26 ± 8.53	21.76–30.24	0.320
	Midfielders	50.94 ± 27.37	37.33–64.56		27 ± 8.51	22.77–31.24	
	Forwards	62.51 ± 25.99	49.59–75.44		31.55 ± 11.8	25.68–37.42	
Defensive Coverage	Defenders	68.51 ± 42.85	35.57–101.45	0.815	37.96 ± 29.64	15.17–60.75	0.638
	Midfielders	81.06 ± 32.07	59.5–102.61		41.74 ± 19.45	28.67–54.81	
	Forwards	81.57 ± 32.84	58.07–105.06		48.79 ± 25.26	30.71–66.86	
Concentration	Defenders	71.66 ± 33.81	54.27–89.05	0.912	29.03 ± 10.28	23.74–34.31	0.620
	Midfielders	79.21 ± 27.32	64.64–93.77		26.58 ± 7.91	22.36–30.8	
	Forwards	77.77 ± 28.22	63.26–92.28		28.05 ± 10.09	22.86–33.24	
Balance	Defenders	64.37 ± 24.69	52.09–76.65	0.561	29.92 ± 13.63	23.14–36.7	0.620
	Midfielders	56.26 ± 26.7	42.98–69.54		32.06 ± 9.27	27.45–36.67	
	Forwards	62.82 ± 24.65	50.55–75.08		29.82 ± 6.64	26.52–33.13	
Defensive Unity	Defenders	62.72 ± 19.26	53.14–72.29	0.361	31.12 ± 7.65	27.31–34.93	0.778
	Midfielders	72.09 ± 23.16	60.57–83.61		33.81 ± 9.54	29.06–38.56	
	Forwards	67.14 ± 21.92	56.24–78.04		31.01 ± 8.32	26.87–35.15	

CI 95%: 95% confidence interval; *p*< 0.05

## Discussion

This study aimed to compare youth male soccer players’ tactical performance according to their positional roles in the field test of FUT-SAT. The results displayed no difference between positional roles in the number of actions, the success percentage, PARP, and TPI. This study indicated that players, regardless of their role, performed all the core tactical principles, thus it was not possible to identify specific tactical behaviour and performance of players in each positional role.

The results of the number of actions corroborated Machado et al. (2019), who found no significant differences in performing tactical actions of core tactical principles in different positional roles. Thus, there was no specificity in tactical behaviour for defenders, midfielders, and forwards in the GK + 3 vs. 3 + GK format. There was no difference because the team configuration was organized with three players for both teams. In an offensive phase, a player in ball possession has only two passing options and the possibility of driving the ball to make feints, move behind, or make the shot. In a defensive phase, players mark players with the ball and off-ball in different field areas according to the ball and opponents' movements. These demands require players to constantly move to several different zones according to the position of the ball, their teammates, and opponents, demonstrating the need to perform all core tactical principles in environments with high variability (da Costa et al., 2011; Duarte et al., 2012; Williams and Hodges, 2005).

Considering the percentage of correct actions and TPI, studies have identified differences in different core tactical principles between players of distinct positional roles with the same experimental design as our study. Regarding the percentage of correct actions, Machado et al. (2019) found that fullbacks presented significantly higher values of the defensive unity and total defensive actions than forwards. With regard to the TPI, Rechenchosky et al. (2017) showed that forwards and midfielders presented significantly higher values than defenders in the offensive unity tactical principle, while forwards and defenders presented significantly higher values in the defensive coverage tactical principle. Finally, Padilha et al. (2013) found significantly higher values in midfielders compared to forwards in the offensive unity tactical principle. Consequently, there is no pattern in the differences between players in tactical performance of different positional roles. Therefore, it is not possible to determine, through the results of the studies, that players in each positional role present specific characteristics of performance of the core tactical principles in the field test of FUT-SAT. Thus, there are indications that it is not possible to identify or select playing positions for each positional role for players through the field test of FUT-SAT, which Costa et al. (2011) determined through the evaluation of tactical behaviour based on core tactical principles. An important point to highlight is that the field test of FUT-SAT ("GK + 3 vs. 3 + GK") did not define a system of play for either team. The system of play is the players' disposition on the field that presents the organization of the team in all sectors (defensive, midfield, offensive) according to their positional role (Bangsbo and Peitersen, 2000). In a game with 11 players, some systems can have at least one and a maximum of six players in each sector, whereas in an SSG with three players, there will be a maximum of only two players in only one of the sectors, which means that the few numbers of players in SSGs do not present the position references of each positional role between players of the same team. Thus, to interact with each other in an organized manner and to achieve common goals in the offensive and defensive phases in SSGs, players of all positional roles must perform all core tactical principles in an effective way (Duarte et al., 2012; Gréhaigne et al., 1999; Hewitt et al., 2016).

Therefore, it is necessary to evaluate soccer players’ tactical performance based on the core tactical principles in games that allow a system of play to be configured according to the offensive and defensive methods of the team, and thus allow the specific evaluation of positional roles. A clear example of that are studies on physical capacities. Studies of positional roles in match play have shown that wide midfielders cover greater distances at speeds of 19.1–23 km/h and >24 km/h than other players, while at speeds of 14.1–19 km/h, central midfielders or wide midfielders were players who covered larger distances (Di Salvo et al., 2007; Lago-Peñas et al., 2009). Owen et al. (2016) showed that in large-sided games within smaller areas (LSGs-Sm) (9 vs. 9 + GK; 45 x 38 yds; 20 min), wide midfielders presented significantly higher RPE values than forwards. Beenham et al. (2017) found that the player workload varied in match play (4-3-3; 90 min), in which midfielders presented significantly higher values of player workload than defenders among elite U17 players. All those studies show that the organization of positional roles in SSGs or match play seeks to indicate the characteristics of players in an environment closer to the reality of the game. Thus, it is relevant to insert the system of play and the game model in the assessment of tactical performance in further studies to understand soccer players’ tactical skills in each positional role and playing position (Dellal et al., 2010).

## Limitations

Some limitations include the fact that teams were organized according to the coach's knowledge. Therefore, the absence of identification of the team’s offensive and defensive game methods, the functions of players in each positional role, and the absence of a system of play with three players are highlighted. Although the duration and the number of bouts of the field test were used to identify all core tactical principles performed by players, some studies have concluded low reliability in tactical behaviour, especially in core tactical principles, which display the necessity to organize an experimental design where players can perform tactical actions with specific functions and roles (Bredt et al., 2016; Clemente et al., 2022; Praça et al., 2022).

## Practical Implications

The field test of FUT-SAT is an experimental design that should be used in training to develop the execution of all tactical principles, especially throughout the season, to enhance players’ performance (Moreira et al., 2021). The tactical evaluation of players in SSGs can help coaches understand what types of activities can stimulate general and specific behaviours for each positional status and identify the tactical skills of each player (Serra-Olivares et al., 2016). Moreover, it is necessary to use games with a greater number of players and space that allow the insertion of a system of play for each positional role that is closer to the reality of match play (Ferreira et al., 2019; Olthof et al., 2019; Pinder et al., 2011). The insertion of the system of play, along with the game ideas, should be introduced in the evaluation of soccer players’ tactical performance to identify each tactical individual demands skill that will be performed in the game.

## Conclusions

The field test of FUT-SAT ("GK + 3 vs. 3 + GK") showed no significant difference in tactical performance between players of different positional roles among young male soccer players. Thestandardfield test of FUT-SAT does not provide evidence that it is a suitable alternative for assessing youth male soccer players’ tactical performance with respect to their roles in the game. Therefore, it is necessary to evaluate teams using a system of play with defined offensive and defensive methods with players in all field sectors in medium or large sided games. In addition, the system of play can be chosen according to the category given that competitions are held in reduced spaces with fewer players.

## Future Research

The study needs to be replicated with medium- or large-sided games with the system of play, and the number of games should be increased to reduce the variability due to contextual factors. Also, research should be conducted on the behaviour of female youth soccer players.

## Data Availability

Not applicable.

## References

[ref1] Aguiar, M., Botelho, G., Lago, C., Maças, V., & Sampaio, J. (2012). A review on the effects of soccer small-sided games. Journal of Human Kinetics, 33, 103–113. 10.2478/v10078-012-0049-x 23486554 PMC3588672

[ref2] Alvero Cruz, J., Cabañas Armesilla, M., Herrero de Lucas, A., Martínez Riaza, L., Moreno Pascual, C., Porta Manzañido, J., Sillero Quintana, M., & Sirvent Belando, J. (2009). Body composition assessment in sports medicine. Statement of spanish group of kinanthropometry of spanish federation of sports medicine. Archivos de Medicina Del Deporte, 131, 166–179.

[ref3] Bangsbo, J., & Peitersen, B. (2000). Soccer systems and strategies. Human Kinetics.

[ref4] Beenham, M., Barron, D. J., Fry, J., Hurst, H. H., Figueirdo, A., & Atkins, S. (2017). A comparison of GPS workload demands in match play and small-sided games by the positional role in youth soccer. Journal of Human Kinetics, 57(1), 129–137. 10.1515/hukin-2017-0054 28713465 PMC5504585

[ref5] Bennett, K. J., Novak, A. R., Pluss, M. A., Stevens, C. J., Coutts, A. J., & Fransen, J. (2018). The use of small-sided games to assess skill proficiency in youth soccer players: A talent identification tool. Science and Medicine in Football, 2(3), 231–236. 10.1080/24733938.2017.1413246

[ref6] Bredt, S. da G. T., Praça, G. M., Figueiredo, L. S., Paula, L. V. de., Silva, P. C. R., Andrade, A. G. P. de., Greco, P. J., & Chagas, M. H. (2016). Reliability of physical, physiological and tactical measures in small-sided soccer Games with numerical equality and numerical superiority. Revista Brasileira de Cineantropometria & Desempenho Humano, 18 10.5007/1980-0037.2016v18n5p602, 602–610.

[ref7] Clemente, F., Aquino, R., Praça, G. M., Rico-González, M., Oliveira, R., Silva, A. F., Sarmento, H., & Afonso, J. (2022). Variability of internal and external loads and technical/tactical outcomes during small-sided soccer games: A systematic review. Biology of Sport, 39(3), 647–672. 10.37766/inplasy2021.3.0080 35959343 PMC9331334

[ref8] Clemente, F. M., Afonso, J., Castillo, D., Los Arcos, A., Silva, A. F., & Sarmento, H. (2020). The effects of small-sided soccer games on tactical behavior and collective dynamics: A systematic review. Chaos, Solitons & Fractals, 134, 109710. 10.1016/j.chaos.2020.109710

[ref9] Clemente, F., & Sarmento, H. (2020). The effects of small-sided soccer games on technical actions and skills: A systematic review. Human Movement, 21(3), 100–119. 10.5114/hm.2020.93014

[ref10] Costa, I. T. da., Garganta, J., Greco, P. J., & Mesquita, I. (2011). Proposal for tactical assessment of Soccer player’s behaviour, regarding core principles of the game. Motriz: Revista de Educação Física, 17(3), 511–524. 10.1590/s1980-65742011000300014

[ref11] Costa, Garganta, J., Greco, P., & Mesquita, I. (2009). Influence kind of ground, size of goalposts and the quantity of the time in the application of the test of ‘GK3-3GK’. Lecturas: Educación Física y Deportes, 14(136), 1.

[ref12] Costa, I., Garganta, J., Greco, P., Mesquita, I., & Maia, J. (2011). System of tactical assessment in Soccer (FUT-SAT): Development and preliminary validation. System, 7(1), 69–83. 10.6063/motricidade.7(1).121

[ref13] Côté, J., & Vierimaa, M. (2014). The developmental model of sport participation: 15 years after its first conceptualization. Science & Sports, 29, S63–S69. 10.1016/j.scispo.2014.08.133

[ref14] da Costa, I. T., da Silva, J. M. G., Greco, P. J., & Mesquita, I. (2009). Tactical principles of Soccer: Concepts and application. Motriz, 15(3), 657–668.

[ref15] Dellal, A., Wong, del P., Moalla, W., & Chamari, K. (2010). Physical and technical activity of soccer players in the French First League-with special reference to their playing position. International SportMed Journal, 11(2), 278–290.

[ref16] Di Salvo, V., Baron, R., Tschan, H., Montero, F. C., Bachl, N., & Pigozzi, F. (2007). Performance characteristics according to playing position in elite soccer. International Journal of Sports Medicine, 28(03), 222–227. 10.1055/s-2006-924294 17024626

[ref17] Dolański, B., Rompa, P., Hongyou, L., Wasielewski, K., & Szwarc, A. (2018). Time-motion characteristics of match-play in elite Polish youth soccer players of various playing positions. Balt J Health Phys Activ, 10(3), 115-123. 10.29359/BJHPA.10.3.13

[ref18] Duarte, R., Araújo, D., Freire, L., Folgado, H., Fernandes, O., & Davids, K. (2012). Intra-and inter-group coordination patterns reveal collective behaviors of football players near the scoring zone. Human Movement Science, 31(6), 1639–1651. 10.1016/j.humov.2012.03.001 22513231

[ref19] Duda H. (2020). Evaluation of football players' actions in individual risky situations of offensive play as a model determinant of a sports game. Journal of Kinesiology and Exercise Sciences, 92(30), 29–39. 10.5604/01.3001.0014.8208

[ref20] Fenner, J. S., Iga, J., & Unnithan, V. (2016). The evaluation of small-sided games as a talent identification tool in highly trained prepubertal soccer players. Journal of Sports Sciences, 34(20), 1983–1990. 10.1080/02640414.2016.1149602 26939880

[ref21] Ferreira, E. C., Belozo, F. L., Grandim, G., Lizana, C., Machado, J. C., Misuta, M., Galatti, L. R., & Scaglia, A. J. (2019). The influence of different game formats in the technical and tactical aspects of soccer players. Revista Brasileira de Educação Física e Esporte, 33(4), 551–560. 10.11606/issn.1981-4690.v33i4p551-560

[ref22] Ford, P. R., Yates, I., & Williams, A. M. (2010). An analysis of practice activities and instructional behaviours used by youth soccer coaches during practice: Exploring the link between science and application. Journal of Sports Sciences, 28(5), 483–495. 10.1016/j.humov.2012.03.001 20419591

[ref23] Garganta, J. (2009). Trends of tactical performance analysis in team sports: Bridging the gap between research, training and competition. Revista Portuguesa de Ciências Do Desporto, 9(1), 81–89. 10.5628/rpcd.09.01.81

[ref24] Gonçalves, E., Rezende, A. L. G. de, & Da Costa, I. T. (2017). Comparison of defensive and offensive tactical performance of U-17 Soccer players from different positions. Revista Brasileira de Ciências do Esporte, 39(2), 108–114. 10.1016/j.rbce.2015.10.015

[ref25] González-Víllora, & Da Costa, I. T. (2015). How to evaluate the soccer tactics? System of Tactical Assessment in Soccer (FUT-SAT). Educación Física y Deporte, 34(2), 467–505. 10.17533/udea.efyd.v34n2a08

[ref26] González-Víllora, S., Serra-Olivares, J., Pastor-Vicedo, J. C., & Da Costa, I. T. (2015). Review of the tactical evaluation tools for youth players, assessing the tactics in team sports: Football. Springerplus, 4(1), 1–17. 10.1186/s40064-015-1462-0 26558166 PMC4630321

[ref27] Gréhaigne, J.-F., Godbout, P., & Bouthier, D. (1999). The foundations of tactics and strategy in team sports. Journal of Teaching in Physical Education, 18(2), 159–174. 10.1123/jtpe.18.2.159

[ref28] Hewitt, A., Greenham, G., & Norton, K. (2016). Game style in soccer: What is it and can we quantify it? International Journal of Performance Analysis in Sport, 16(1), 355–372. 10.1080/24748668.2016.11868892

[ref29] Hill-Haas, S. V., Dawson, B., Impellizzeri, F. M., & Coutts, A. J. (2011). Physiology of small-sided games training in football: A systematic review. Sports Medicine, 41(3), 199–220. 10.2165/11539740-000000000-00000 21395363

[ref30] Lago-Peñas, C., Rey, E., Lago-Ballesteros, J., Casais, L., & Dominguez, E. (2009). Analysis of work-rate in soccer according to playing positions. International Journal of Performance Analysis in Sport, 9(2), 218–227. 10.1080/24748668.2009.11868478

[ref31] Machado, G., Bach Padilha, M., González Víllora, S., Clemente, F. M., & Teoldo, I. (2019). The effects of positional role on tactical behaviour in a four-a-side small-sided and conditioning soccer game. Kinesiology, 51(2), 261–270. 10.26582/k.51.2.15

[ref32] McKay, A. K., Stellingwerff, T., Smith, E. S., Martin, D. T., Mujika, I., Goosey-Tolfrey, V. L., Sheppard, J., & Burke, L. M. (2022). Defining training and performance caliber: A participant classification framework. International Journal of Sports Physiology and Performance, 17(2), 317–331. 10.1123/ijspp.2021-0451 34965513

[ref33] Moreira, P., Sousa, R. B., Morales, J. C. P., Greco, J. P., Arroyo, M. P. M., & Praça, G. M. (2021). Tactical behaviour of soccer players from different playing positions throughout a season. Retos: Nuevas Tendencias En Educación Física, Deporte y Recreación, 39, 1–6. 10.47197/retos.v0i39.75970

[ref34] Olthof, S. B., Frencken, W. G., & Lemmink, K. A. (2019). A match-derived relative pitch area facilitates the tactical representativeness of small-sided games for the official soccer match. Journal of Strength and Conditioning Research, 33(2), 523. 10.1519/jsc.0000000000002978 30550401 PMC6358197

[ref35] Owen, A. L., Dunlop, G., Rouissi, M., Haddad, M., Mendes, B., & Chamari, K. (2016). Analysis of positional training loads (ratings of perceived exertion) during various-sided games in European professional soccer players. International Journal of Sports Science & Coaching, 11(3), 374–381. 10.1177/1747954116644064

[ref36] Padilha, M. B., Moraes, J. C., & da Costa, I. T. (2013). Can positional statute influence tactical performance of U-13 youthsoccer players?. Revista Brasileira de Ciência e Movimento, 21(4), 73–79. 10.18511/0103-1716/rbcm.v21n4p73-79

[ref37] Pinder, R. A., Davids, K., Renshaw, I., & Araújo, D. (2011). Representative learning design and functionality of research and practice in sport. Journal of Sport and Exercise Psychology, 33(1), 146–155. 10.1123/jsep.33.1.146 21451175

[ref38] Praça, G. M., Abreu, C. de O., Rochael, M., & Moreira, P. D. (2022). How reliable are the tactical measures obtained in soccer small-sided games? A test-retest analysis of observational instruments and GPS-based variables. Proceedings of the Institution of Mechanical Engineers, Part P: Journal of Sports Engineering and Technology, 00(0), 1–10. 10.1177/17543371221113925

[ref39] Radziminski L., Szwarc A., Jastrzebski Z., Rzeszutko-Belzowska A. Relationships between technical and physical match performance in elite soccer. Balt J Health Phys Act. 2022;14(4):Article1. 10.29359/BJHPA.14.4.01

[ref40] Rechenchosky, L., Borges, P. H., Menegass, V. M., de Oliveira Jaime, M., Guilherme, J., Teoldo, I., & Rinaldi, W. (2017). Comparison of tactical principles efficiency among soccer players from different game positions. Human Movement Special Issues, 2017(5), 31–38. 10.1515/humo-2017-0040

[ref41] Robinson, G., & O’Donoghue, P. (2007). A weighted kappa statistic for reliability testing in performance analysis of sport. International Journal of Performance Analysis in Sport, 7(1), 12–19. 10.1080/24748668.2007.11868383

[ref42] Sarmento, H., Clemente, F. M., Harper, L. D., Costa, I. T. da., Owen, A., & Figueiredo, A. J. (2018). Small sided games in soccer–a systematic review. International Journal of Performance Analysis in Sport, 18(5), 693–749. 10.1080/24748668.2018.1517288

[ref43] Serra-Olivares, J., Clemente, F. M., & González-Víllora, S. (2016). Tactical expertise assessment in youth football using representative tasks. Springerplus, 5(1), 693–749. 10.1186/s40064-016-2955-1 27547675 PMC4978649

[ref44] Szwarc, A., Dolanski, B., & Lipinska, P. (2015). The set of test tasks assessing special physical fitness of 17-year-old soccer players. Balt J Health Phys Activ, 7, 51-58. 10.29359/BJHPA.07.1.05

[ref45] Tabachnick, B. G. & Fidell, L. S. (2012). *Using multivariate statistics* (6th ed.). Pearson.

[ref46] Thomas, J. R., Martin, P., Etnier, J. L., & Silverman, S. J. (2022). Research methods in physical activity. Human Kinetics.

[ref47] Ward, P., Hodges, N. J., Starkes, J. L., & Williams, M. A. (2007). The road to excellence: Deliberate practice and the development of expertise. High Ability Studies, 18(2), 119–153. 10.1080/13598130701709715

[ref48] Williams, A. M., & Hodges, N. J. (2005). Practice, instruction and skill acquisition in soccer: Challenging tradition. Journal of Sports Sciences, 23(6), 637–650. 10.1080/02640410400021328 16195012

[ref49] World Medical Association. (2013). World Medical Association Declaration of Helsinki: Ethical Principles for Medical Research Involving Human Subjects. JAMA, 310(20), 2191–2194. 10.1001/jama.2013.281053 24141714

